# The relationship between sarcopenia detected in newly diagnosed lung cancer patients and serum levels of IL6, IGF-1 and myostatin

**DOI:** 10.3389/fragi.2026.1800359

**Published:** 2026-08-06

**Authors:** Kamil Süzer, Utku Oflazoglu, Leyla Demir, Sinan Unal, Zeynep Gulsum Guc, Yasar Yildiz, Furkan Oguzhan Karalar, Yuksel Kucukzeybek, Ahmet Alacacioglu

**Affiliations:** 1 Department of Internal Medicine, Ataturk Training and Research Hospital, Izmir Katip Celebi University, Izmir, Türkiye; 2 Department of Medical Oncology, Ataturk Training and Research Hospital, Izmir Katip Celebi University, Izmir, Türkiye; 3 Department of Biochemistry, Ataturk Training and Research Hospital, Izmir Katip Celebi University, Izmir, Türkiye

**Keywords:** bio-electric impedance analysis, IGF-1, IL-6, lung cancer, myostatin, sarcopenia

## Abstract

**Aim:**

Sarcopenia can result from many factors, including cancer, and is a consequence of quantitative and qualitative deterioration in skeletal muscle mass. Although there are some hypothetical explanations for sarcopenia, the underlying mechanisms of this condition have not been clearly defined in cancer patients. In this study, we aimed to investigate the association between sarcopenia and serum levels of myostatin, insulin-like growth factor-1 (IGF-1), and interleukin-6 (IL-6) in patients with newly diagnosed lung cancer.

**Material and Method:**

This cross-sectional prospective study included patients with newly diagnosed metastatic non-small cell lung cancer (NSCLC), who were categorized into two groups based on the presence or absence of sarcopenia. Body composition and muscle mass were evaluated using bioelectrical impedance analysis (BIA), and body mass index (BMI) was calculated. Handgrip strength was measured using a handheld dynamometer. Serum levels of myostatin, insulin-like growth factor-1 (IGF-1), and interleukin-6 (IL-6) were analyzed from fasting venous blood samples to assess their association with sarcopenia.

**Results:**

A total of 69 patients were included in the study. The patients’ ages ranged from 49 to 75 years. Thirty-four of these patients were sarcopenic and thirty-five of them were non-sarcopenic. Eighty-seven percent (60) of the patients included in the study were male. While myostatin and IL-6 levels were higher in the sarcopenic patient group, IGF-1 mean levels were lower. In the non-sarcopenic patient group, the median levels of myostatin, IL-6, and IGF-1 were 11.82 ng/mL, 1.17 pg/mL and 23.51 ng/mL, respectively. In the sarcopenic patient group, the median levels of myostatin, IL-6, and IGF-1 were 16.76 ng/mL, 7.42 pg/mL and 12.82 ng/mL, respectively (p < 0.001, p < 0.01, and p < 0.001, respectively). Spearman’s correlation analysis between SMI (skeletal muscle mass index) and myostatin, IGF-1 and IL-6 levels showed a negative correlation of SMI with myostatin and IL-6, and a positive correlation with IGF-1 [(r = −0.436, p < 0.001) (r = −0.520, p < 0.001), (r = 0.219, p:0.071) respectively]. When a logistic regression model was constructed with sarcopenia status as the dependent variable and myostatin, IGF-1, IL-6, and ECOG performance parameters as independent variables, myostatin and IGF-1 were identified as independent predictors [OR: 1.332, CI: (1.128–1.574), p: 0.001; OR: 0.926, CI: (0.874–0.982), p: 0.01, respectively].

**Conclusion:**

We found a significant correlation between sarcopenia and myostatin, IGF-1 and IL-6 in patients with lung cancer. We suggest that myostatin, IGF-1, and potentially IL-6 may serve as reliable markers for sarcopenia in cancer patients. We believe that these findings may encourage future prospective studies investigating the potential relevance of myostatin, IGF-1 and IL-6 as markers of sarcopenia.

## 1 Introduction

Sarcopenia is a progressive syndrome characterized by a decline in muscle mass, muscle strength, and physical performance, as defined by the European Society for Clinical Nutrition and Metabolism (ESPEN) and the European Working Group on Sarcopenia in Older People (EWGSOP) (Cederholm et al., 2017; Cruz-Jentoft et al., 2019). The incidence of sarcopenia increases with age and is particularly significant for cancer patients (US Cancer Statistics Working Group, 2013). In patients with malignancy, muscle loss is associated with major morbidities such as tumor progression, increased chemotherapy-induced toxicity, and prolonged hospitalization (Peterson and Mozer, 2017). A strong correlation has been identified between sarcopenia and lung cancer; notably, a high incidence of sarcopenia has been observed in patients with advanced-stage lung cancer (Kimura et al., 2015; Baracos et al., 2010). Diagnosis of sarcopenia requires a decline in muscle strength, reduced muscle quality and quantity (mass), and/or impaired physical performance (Dent et al., 2018). According to the definition of sarcopenia updated by EWGSOP in 2018, low muscle strength is defined as ‘probable sarcopenia’; the additional presence of low muscle quality and quantity (mass) confirms ‘confirmed sarcopenia,’ and the concomitant presence of all three criteria is defined as ‘severe sarcopenia’ (Cruz-Jentoft et al., 2019).

Muscle quantity or mass is measured using various methods such as dual-energy X-ray absorptiometry (DXA), bioelectrical impedance analysis (BIA), computed tomography (CT), and magnetic resonance imaging (MRI). Although each method has its own advantages and disadvantages (Cho et al., 2022), BIA is frequently utilized in clinical studies for body composition assessment due to its non-invasive nature, ease of use, relative cost-effectiveness, and low operator-dependent error (González-Correa and Caicedo-Eraso, 2012).

In addition to the measurement of muscle mass and muscle strength, various serum biomarkers related to sarcopenia are being developed. These include numerous adipomyokines secreted by skeletal myocytes or adipocytes, such as muscle growth inhibitors (interleukin-6 [IL-6], myostatin, activins A/B, growth and differentiation factor 15) and muscle growth promoters (follistatin, irisin, bone morphogenetic proteins [BMPs]), as well as brain-derived neurotrophic factor (Pedersen, 2011a; Pedersen, 2011b; Pedersen et al., 2007; Halmos and Suba, 2014).

Myostatin is a member of the TGF-β superfamily that acts as the primary negative regulator of skeletal muscle growth during embryonic and postnatal development in animals (Durieux et al., 2007; McPherron et al., 1997) and humans (McPherron and Lee, 1997), as well as in adulthood (Schuelke et al., 2004). This myokine functions as a direct negative regulator of muscle growth through activin type II receptors and SMAD2-3 transcription factors (Lee, 2021). Myostatin, TGF-β, and bone morphogenetic proteins are the most extensively characterized ligands regarding their effects on skeletal muscle. Myostatin produced by the muscle inhibits muscle anabolism and satellite cell production, and it has been associated with loss of muscle mass (Esposito et al., 2022). There is evidence regarding increased myostatin levels in the pathogenesis of age-associated loss of muscle mass, cachexia associated with human immunodeficiency virus and cachexia associated with cancer (Yarasheski et al., 2002; Gonzalez-Cadavid et al., 1998; Zimmers et al., 2002; Costelli et al., 2008). Alterations in myostatin expression in skeletal and cardia muscle following exercise training have also been reported (Lenk et al., 2009).

While myostatin gene knockout in mice leads to muscle hypertrophy, transgenic mice with overexpression of myostatin in skeletal muscle exhibit muscle atrophy (Lee, 2021). Consistent with these data, several studies have suggested that myostatin may contribute to muscle wasting in humans (Breitbart et al., 2011; Breitbart et al., 2013). However, recent studies have found a positive correlation between circulating myostatin and muscle mass, suggesting that this myokine could serve as a biomarker of muscle mass (Alexopoulos et al., 2021; Nishikawa et al., 2022). Consequently, myostatin has become one of the primary targets in the investigation of muscle mass regulation.

Myostatin also inhibits the insulin/IGF-1–AKT–mTOR pathway (Amirouche et al., 2009). One of the primary positive regulators of muscle growth is insulin-like growth factor 1 (IGF-1). Under normal conditions, IGF-1 signaling appears to be dominant and blocks the myostatin pathway (Trendelenburg et al., 2009). However, inhibition of IGF-1 has been observed when myostatin is overexpressed (Amirouche et al., 2009; Morissette et al., 2009). Available data indicate that the activity of this pathway is diminished in the muscles of sarcopenic patients. In cachectic cancer patients, circulating IGF-1 (Simons et al., 1999) and muscle IGF1-Ec transcript levels (Bonetto et al., 2013) are reduced. Furthermore, studies in cachectic cancer animal models have demonstrated that circulating IGF-1 levels (Costelli et al., 2006; Huot et al., 2020; Koutnik et al., 2020), as well as muscle IGF1-Ea (Costelli et al., 2006) and IGF1-Eb (White et al., 2011; Constantinou et al., 2011) transcript levels and muscle IGF-1 protein content (Zhuang et al., 2016), are decreased.

IL-6 is a proinflammatory cytokine that normally maintains the balance between synthesis and catabolism in skeletal muscle. Elevated levels of proinflammatory cytokines lead to increased catabolism, inhibition of protein synthesis, impairment of muscle integrity and function, and ultimately, sarcopenia (Pan et al., 2021). Furthermore, increased IL-6 levels contribute to the progression of sarcopenia (Krabbe et al., 2004; Franceschi and Campisi, 2014). According to a recent meta-analysis, serum levels of IL-6 are negatively correlated with muscle strength in healthy elderly populations (Mikó et al., 2018).

Investigating the pathophysiology of sarcopenia, ensuring early diagnosis, and preventing its development—particularly in cancer patients—are of paramount importance. This allows patients to remain physically stronger and improves their adherence to treatment. Based on our literature review, serum IL-6, myostatin, and IGF-1 levels have not been previously investigated together in patients with lung cancer-associated sarcopenia. The aim of the present study is to identify the factors affecting the development of sarcopenia in patients with newly diagnosed lung cancer, as well as to evaluate the use of novel molecules in the detection of sarcopenia and to gain a deeper insight into its pathophysiology. Additionally, this study aims to contribute to future efforts to improve quality of life, overall survival, and treatment complications by enabling early detection of sarcopenia in this patient group.

## 2 Materials and methods

### 2.1 Patients and study design

The present study included patients diagnosed with non-small cell lung cancer (NSCLC) admitted to the Department of Medical Oncology at Izmir Katip Celebi University (IKCU) Ataturk Training and Research Hospital between October 2023 and August 2024. Based on predefined criteria, 69 patients were eligible for analysis. Inclusion criteria comprised patients aged 18–75 years, with adequate cognitive, liver, and renal functions, and no history of anti-inflammatory or beta-blocker therapy, or oral enteral nutritional support.

To ensure the integrity of the biomarker assessments, we strictly excluded patients with a history of major surgery or trauma within the preceding 3 months, as well as those with multiple organ dysfunction. These factors were meticulously controlled through a comprehensive review of electronic health records and detailed clinical anamnesis obtained during enrollment. Furthermore, only newly diagnosed patients with no signs of active infection at the time of blood sampling and who provided written informed consent were included in the study.

### 2.2 Assessment of body composition

Body composition was assessed using a TANITA SC-330 bioelectrical impedance analysis (BIA) device. BIA is an analytical method based on the differences in electrical conductivity between fat-free mass and body fat. Bioelectrical impedance was measured at 800 mA using a BIA device with an operating frequency of 50 kHz. The electrical and biological parameters measured by BIA vary among individuals. This device is capable of reporting more than 20 parameters, including bioelectrical resistance, fat mass, body fat percentage, visceral fat rating, and body mass index (BMI). As part of the study, subjects were asked to stand with both feet, ensuring their heels and soles were positioned on two metallic electrodes on the scale. The analysis proceeded by evaluating the bioimpedance of a 50 kHz electrical current transmitted through the body via the electrodes placed under the feet (Engin et al., 2014). Once the measurements stabilized, the analyzer displayed the resistance directly and immediately. For the calculation of absolute skeletal muscle mass (SM), we relied on the methodology described in previous studies (Janssen et al., 2000; Baumgartner et al., 1998). The BIA equation was utilized for the calculation of skeletal muscle mass.

Skeletal muscle mass was calculated by measuring impedance via BIA using the following formula:

$$Skeletal\;Muscle\;Mass\;\left(kg\right)=[0.401\times \left({Height}^{2}/Resistance\right)+\left(3.8\times Gender\right)-\left(0.071\times Age\right)+5.102]$$

In this equation, height is expressed in centimeters (cm), resistance in ohms (Ω), and gender is coded as 1 for males and 0 for females. To convert the absolute skeletal muscle mass into the Skeletal Muscle Index (SMI), the values were divided by the square of the height in meters (m^2^) (Janssen et al., 2000; Baumgartner et al., 1998). To ensure measurement standardization, all assessments were conducted in a temperature-controlled room (22 °C–24 °C). Participants were required to refrain from food and fluid intake for at least 3 h prior to the procedure. Measurements were taken while patients were wearing light clothing, and had removed their shoes, socks, and any metallic accessories, in accordance with the manufacturer’s protocol.

### 2.3 Assessment of muscle strength

Muscle strength was evaluated using a calibrated manual handgrip dynamometer (TAKEI 5401, Takei Scientific Instruments Co., Ltd., Niigata, Japan; capacity 100 kg). To ensure optimal and reproducible test results, participants were instructed to perform three maximal isometric contractions using their dominant hand, with a minimum recovery interval of two minutes between attempts. The peak force, defined as the highest value obtained across the three repetitions, was recorded in kilograms (kg) for subsequent analysis.

### 2.4 Body mass index (BMI)

Standard measurement procedures were utilized for height and weight assessments; patients wore light clothing and removed their shoes during the measurements. Height and weight were measured with a precision of 0.1 cm and 0.1 kg, respectively. Body Mass Index (BMI) was calculated using the following formula: BMI = weight (kg)/height^2^ (m^2^). BMI categories typically applied to adults were employed to classify the patients as follows: <20.0 kg/m^2^, underweight; 20.0–24.9 kg/m^2^, normal weight; 25.0–29.9 kg/m^2^, overweight; and >30.0 kg/m^2^, obese (WHO, 2025).

### 2.5 Definition of sarcopenia and cut-off values

The diagnosis of sarcopenia was established based on the EWGSOP consensus (Cruz-Jentoft et al., 2019; Cr et al., 2010). A review of the literature revealed that sarcopenia threshold values for patients with cancer have not been previously defined. As sarcopenia studies indicate that the threshold values for sarcopenia used in patients with cancer are based on those established for non-cancer patients, the cut-off values were determined according to the skeletal muscle mass index (SMI) and handgrip strength values recommended by the European Working Group on Sarcopenia in Older People (EWGSOP). The gender-specific threshold values for sarcopenia are as follows: a low SMI is defined as < 10.76 kg/m2, for men and <6.76 kg/m2 for women (Cruz-Jentoft et al., 2019). Additionally, low handgrip strength is set at <27 kg for men and <16 kg for women (Cr et al., 2010). Sarcopenia is defined as the coexistence of low skeletal muscle mass and low muscle strength.

### 2.6 Biochemical analysis

Venous blood samples were collected from all patients between 08:00 and 09:00 a.m., following an 8–12 h fasting, while the patients were in a seated position. The blood was drawn into gel-containing serum separator tubes (BD Vacutainer SST, 8.5 mL). The tubes were placed vertically in a rack allowing the samples to clot for 30 min at room temperature (24 °C), and then centrifuged at 2,000 *g* for 10 min. The separated serum samples were then portioned into capped Eppendorf tubes and stored at −20 °C until the time of analysis.

Serum Myostatin, IL-6, and IGF-1 levels were determined using enzyme-linked immunosorbent assay (ELISA) kits. After thawing at room temperature, the serum samples were vortexed to ensure homogenization of precipitated protein molecules. The assays were performed in strict accordance with the procedures provided in the ELISA kits. The tests, which utilized the sandwich ELISA method, were read at 450 nm using a microplate reader (Biotek ELx800, USA), and the results were calculated using a standard curve.

Serum Myostatin (Catalog No: E-EL-H1437), IL-6 (Catalog No: E-EL-H6156), and IGF-1 (Catalog No: E-EL-H0086) levels were analyzed using Elabscience (Houston, USA) ELISA kits with a coefficient of variation (CV) of <10%.

### 2.7 Statistical analysis

Continuous variables were expressed as mean and standard deviation, while categorical variables were presented as numbers and percentages. Numerical variables were evaluated using the Kolmogorov-Smirnov test and histograms to determine whether they exhibited a normal or skewed distribution. Normally distributed continuous data were compared using the independent samples t-test, whereas non-normally distributed continuous data were compared using the Mann-Whitney U test.

Categorical data were analyzed using the Fisher’s Exact test or the Chi-square test. A univariate analysis was performed to identify potential risk factors for sarcopenia. Variables with a p-value <0.05 in the univariate analysis were subsequently included in a multivariate forward logistic regression analysis.

Before constructing the logistic regression model, the underlying assumptions were tested. Model fit was assessed using the Hosmer–Lemeshow test, and the model was found to fit the data well (p > 0.05). The explanatory power of the model was reported using the Nagelkerke *R* ^2^ value. Multicollinearity among the independent variables was assessed by examining VIF values, and no multicollinearity was identified (VIF <5). Additionally, the linearity of the logit for continuous variables was tested using the Box–Tidwell method, and the assumption was found to be satisfied.

Correlations were analyzed using Spearman’s correlation method. Linear and logistic regression equations were also employed for the analysis. Logistic regression analysis was performed to estimate the odds ratios (OR) and 95% confidence intervals (CI) for sarcopenia. A p-value <0.05 was considered statistically significant. Statistical analyses were conducted using SPSS software (version 20.0, SPSS Inc., Chicago, IL, 2018).

### 2.8 Sample size and power analysis

Since no previous study has investigated newly diagnosed cancer patients in this context, we estimated the number of patients to be recruited during the planned one-year study period based on the current definitions and measurement methods of sarcopenia in cancer patients previously published by Oflazoglu et al. In our previous study, the incidence of sarcopenia in newly diagnosed lung cancer patients was found to be 13.8%. In that study, 29 out of 467 cancer patients diagnosed in the last year had lung cancer, and the sarcopenia rate in this subgroup was 13.9% (Oflazoglu et al., 2020). In this study, to equalize the number of patients in both groups and ensure homogeneity, we maintained an equal ratio of sarcopenic to non-sarcopenic patients. When the first mean was entered as 13.8% and the second as 50% in the G*Power software package, the effect size was calculated as 0.76 with 80% power and a 0.05 significance level. Accordingly, we calculated that a minimum sample size of at least 26 patients per group, for a total of 52 patients, would be required. Since our study was designed as a pilot study and represents the first of its kind in the Turkish population, we estimated that the expected number of patients during the study period would be at least 26 per group. In the *post hoc* sample analysis performed after completion of the study, the IL-6 value was 19.94 (SD: 29.47) in sarcopenic patients and 4.51 (SD: 9.36) in non-sarcopenic patients. Using these values, a sample size calculation was performed with the G*Power software package with 80% power and a 0.05 margin of error, yielding an effect size of 0.71 and indicating that at least 32 patients per group would be required.

#### 2.8.1 Ethics

This study was submitted to the Izmir Katip Celebi University Ataturk Training and Research Hospital Invasive Scientific Research Ethics Committee and received approval on 9 November 2023, with Decision No: 0065 (09/11/2023–0065). All procedures performed in this study were in accordance with the ethical standards of the institutional research committee and the 1964 Helsinki Declaration and its later amendments.

## 3 Results

In our study, a total of 80 patients were screened. Patient enrollment continued until at least 30 patients were included in each group. Initially, 80 patients were planned to be included in this cross-sectional prospective study; however, four patients refused to allow blood samples to be taken after measurements were completed, 1 patient died before starting treatment (due to pulmonary embolism), and 3 patients withdrew their consent to participate in the study. In addition, two more patients were excluded from the study after measurements were taken due to medication use identified according to the study exclusion criteria. Therefore, a total of 11 patients were excluded from the analysis. Finally, a total of 69 patients were included in the study, consisting of 34 sarcopenic and 35 non-sarcopenic individuals. Of the patients, 9 (13%) were female and 60 (87%) were male. The number of patients with a known history of diabetes was 15 (21.7%). Regarding performance status, 45 patients (65.2%) had an ECOG performance score of 0. Furthermore, 15.9% (n = 11) of the patients had undergone curative surgery for their primary tumor. Approximately half of the patients (49.2%, n = 34) were under the age of 65. The number of patients with albumin levels ≥3.5 g/dL was 60 (86.9%). When categorized according to Body Mass Index (BMI), 42.1% (n = 29) of the patients were classified as obese or overweight, while 57.9% (n = 40) were normal or underweight. Table 1 summarizes the demographic characteristics of the patients overall and according to their sarcopenic status.

**TABLE 1:** Relationship between participant and disease characteristics according to sarcopenic status.

*Variables*	*Non-sarcopenic n (%)*	*Sarcopenic n (%)*	*P value*
All patients	35 (100%)	34 (100%)	​
Sex	​	​	p:0.734
Male	31 (88.5%)	29 (85.3%)	​
Female	4 (11.5%)	5 (14.7%)	
Operation	​	​	p:0.752
Yes	5 (14.2%)	6 (17,6%)	​
No	30 (85.8%)	28 (82,4%)	
BMI (kg/m^2)^	​	​	p:0.809
BMI <25	21 (60%)	19 (55.8%)	​
BMI ≥25	14 (40%)	15 (44.2%)	​
ECOG performance status	​	​	**p:0.045**
0	27 (77.1%)	18 (52.9%)	​
1 and 2	8 (22.9%)	16 (47.1%)	​
Age (years)	​	​	p:0.473
<65	19 (54.2%)	15 (44.2%)	​
≥65	16 (45.8%)	19 (55.8%)	​
Diabetes	​	​	p:0.394
Yes	6 (17.1%)	9 (26.4%)	​
No	29 (82.9%)	25 (73.6%)	​
Albumin (gr/dL)	​	​	p:0.084
<3.5	2 (5.7%)	7 (20.5%)	​
≥3.5	33 (94.3%)	27 (79.5%)	​

ECOG, eastern cooperative oncology group, BMI, body mass index, p-values <0.05 are indicated in bold.

An examination of the factors potentially associated with sarcopenia (including age, gender, BMI, ECOG performance score, diabetes, albumin levels, and history of curative surgery) showed that only a poor performance status (ECOG >0) was significantly associated with a higher frequency of sarcopenia (p = 0.045). The relationships among age, skeletal muscle index (SMI), handgrip strength (HGS), height, weight, and albumin levels in non-sarcopenic versus sarcopenic patients are summarized in Table 2.

**TABLE 2:** Clinical characteristics of all patients categorized according to sarcopenic status.

*Variables*	*Non-sarcopenic (*mean/SD)	*Sarcopenic (*mean/SD*)*	*P value*
All patients (n)	35	34	​
Age (years)	65.14 (SD:9.28)	67.18 (SD:10.23)	0.517
SMI (cm^2^/m^2^)	11.13 (SD:1.79)	8.42 (SD:1.53)	**<0.001**
HGS (kg)	33 (SD:8.15)	21.61 (SD:5.60)	**<0.001**
Height (cm)	171.08 (SD:8.17)	168.64 (SD:7.71)	0.152
Weight (kg)	73.79 (SD:12.53)	68.29 (SD:14.76)	0.211
Albumin (g/dL)	4.01 (SD:0.33)	3.77 (SD:0.42)	**0.029**

SD: standard deviation; SMI: skeletal muscle mass index, HGS: handgrip strength, IL-6: Interleukin-6, IGF-1: Insulin-like growth factor-1, p-values <0.05 are indicated in bold.

In the non-sarcopenic patient group, the median serum levels of myostatin, IL-6, and IGF-1 were 11.82 ng/mL, 1.17 pg/mL, and 23.51 ng/mL, respectively. In contrast, in the sarcopenic patient group, the mean serum levels of myostatin, IL-6, and IGF-1 were found to be 16.76 ng/mL, 7.42 pg/mL, and 12.82 ng/mL, respectively (p < 0.001, p < 0.01, and p < 0.001, respectively) (Table 3).

**TABLE 3:** Myostatin, IGF-1, and IL-6 levels according to sarcopenic status.

*Variables (mean, SD)*	*Myostatin (ng/mL)*	*p-value* *	*IGF-1 (ng/mL)*	*p-value* *	*IL-6 (pg/mL)*	*p-value* *
All patients (n:69)	​	*p < 0*.*001*	​	*p < 0*.*001*	​	*p < 0*.*01*
*- Sarcopenic (n:34)*	*16.76 (7.3–69.9)*	​	*12.82 (0.57–60.1)*	​	*7.42 (0.3–117)*	​
*- Non sarcopenic (n:35)*	*11.82 (2.46–22.2)*		*23.5 (3.85–147.4)*		*1.17 (0.29–48.71)*	

* The comparison of sarcopenic patients and non-sarcopenic patients in terms of inflammatory markers was performed using Mann-Whitney U test.

SD, standard deviation, IL-6, Interleukın-6, IGF-1, Insulin-like growth factor-1, p-values <0.05 are indicated in bold.

Spearman’s correlation analysis between SMI and serum levels of myostatin, IGF-1, and IL-6 indicated a negative correlation of SMI with myostatin and IL-6, and a positive correlation with IGF-1 [(r = −0.436, p < 0.001) (r = −0.520, p < 0.001), and (r = 0.219, p = 0.071), respectively]. However, while the correlations between SMI and myostatin and IL-6 were statistically significant, no statistically significant correlation was found between SMI and IGF-1. Similarly, in the Spearman’s correlation analysis conducted between HGS and these biomarkers, a negative correlation was identified between HGS and myostatin and IL-6, whereas a positive correlation was found with IGF-1 serum levels [(for myostatin: r = −0.405, p = 0.001) (for IL-6: r = −0.363, p = 0.002), and (for IGF-1: r = 0.254, p = 0.035)] (Table 4).

**TABLE 4:** Spearman’s correlation analysis of SMI, HGS, and related factors.

*Myostatin level*			IGF-1 level		IL-6 level	
All patients (n:69)	r	p value	r	p value	r	p value
*- SMI*	−0.436	<0.001	0.219	0.071	−0.520	<0.001
*- HGS*	−0.405	0.001	0.254	0.035	−0.363	0.002

SMI, skeletal muscle mass index, HGS, handgrip strength, IL-6: Interleukın-6, IGF-1, Insulin-like growth factor-1, p-values <0.05 are indicated in bold.

In the logistic regression analysis, with sarcopenia as the dependent variable and myostatin, IGF-1, IL-6, and ECOG performance status as independent variables, both myostatin and IGF-1 were identified as independent predictors of sarcopenia [For myostatin: OR = 1.332, 95% CI: (1.128–1.574), p = 0.001; for IGF-1: OR = 0.926, 95% CI (0.874–0.982), p = 0.01; for IL-6: OR = 1.057, 95% CI: (0.992–1.125), p = 0.086] (Table 5).

**TABLE 5:** Logistic regression analysis of sarcopenia and related factors.

Variables	*β*	*SE*	Normal range	OR	p value
*-Constant*	−2.170	1.159	​	0.114	0.061
*-Myostatin*	0.287	0.085	1.128–1.574	1.332	**0.001**
*-IL-6*	0.055	0.032	0.992–1.125	1.057	0.086
*-IGF-1*	−0.077	0.030	0.874–0.982	0.926	**0.010**
*-ECOG 0/*1 and 2	−0.869	0.797	0.088–2.000	0.419	0.276

β, Regression coefficient; SE, standard error; OR, odds ratio; p-values <0.05 are indicated in bold. IL-6, Interleukin-6; IGF-1, Insulin-like Growth Factor-1; ECOG, Eastern Cooperative Oncology Group. Hosmer-Lemeshov test p value:0.798, Nagelkerke R square: 0.609.

When simple linear regression analysis was performed between HGS and serum levels of myostatin, IGF-1, and IL-6, statistical significance was observed for myostatin and IL-6 (*R* ^*2*^ = 0.206, p = 0.031 and *R* ^*2*^ = 0.279, p = 0.020 respectively) (Table 6).

**TABLE 6:** Linear regression analysis between HGS and Myostatin, IGF-1, and IL-6 levels.

Variables*	β	*SE*	*t*	*R* ^*2*^	*p value*
*- Constant*	32.123	1.980	16.222	0.324	<0.001
*- Myostatin*	−0.307	0.110	−2.803	​	**0.007**
*- Constant*	28.006	1.492	18.767	0.074	<0.001
*- IGF-1*	−0.024	0.039	−0.604	​	0.548
*- Constant*	28.722	1.190	24.144	0.279	<0.001
- *IL-6*	−0.110	0.046	−2.382	​	**0.020**

* Simple linear regression analysis.

β, Unstandardized coefficient; SE, standard error; *R* ^*2*^: coefficient of determination, IL-6, Interleukin-6; IGF-1, Insulin-like Growth Factor-1, p-values <0.05 are indicated in bold.

In the simple linear regression analysis conducted between SMI and serum levels of myostatin, IGF-1, and IL-6, statistical significance was observed only for myostatin (*R* ^*2*^ = 0.206, p = 0.031) (Table 7).

**TABLE 7:** Linear regression analysis between SMI and Myostatin, IGF-1, and IL-6 levels.

Variables*	β	*SE*	*t*	*R* ^*2*^	p value
*- Constant*	10.704	0.481	22.269	0.261	<0.001
*- Myostatin*	−0.059	0.027	−2.209	​	**0.031**
*- Constant*	9.907	0.355	27.874	0.054	<0.001
*- IGF-1*	−0.004	0.009	−0.445	​	0.658
*- Constant*	10.032	0.288	34.788	0.206	<0.001
*- IL-6*	−0.019	0.011	−1.723	​	0.090

* Simple linear regression analysis.

β, Unstandardized coefficient; SE, standard error; *R* ^*2*^, coefficient of determination, IL-6, Interleukin-6; IGF-1, Insulin-like Growth Factor-1, p-values <0.05 are indicated in bold.

## 4 Discussion

In this cross-sectional, prospective study investigating the relationship between sarcopenia and serum levels of myostatin, IGF-1, and IL-6 in patients with newly diagnosed lung cancer, sarcopenic patients were found to have higher serum levels of myostatin and IL-6 and, conversely, lower serum levels of IGF-1 compared to non-sarcopenic patients. When factors associated with sarcopenia were examined, univariate analysis revealed that an ECOG performance score >0, low IGF-1 serum levels, and high IL-6 and myostatin serum levels were significantly associated with sarcopenia. Furthermore, logistic regression analysis showed that myostatin and IGF-1 were independent predictors of sarcopenia.

Specifically, IL-6 acts as an important mediator in muscle mass loss by affecting various muscle catabolic and anabolic pathways (Madeddu et al., 2015; Muñoz-Cánoves et al., 2013). According to a meta-analysis by Miko et al. including 26 studies, serum IL-6 levels show a negative correlation with muscle strength in healthy elderly populations (Mikó et al., 2018). A five-year prospective cohort study by Aleman et al. linking cytokine levels to muscle mass concluded that high serum IL-6 and CRP levels were significantly associated with total appendicular skeletal muscle loss (Alemán et al., 2011). In a prospective study of 74 patients with NSCLC conducted by Park et al., thirteen cytokines and myokines were evaluated. The levels of key cytokines associated with poor prognosis—including IL-6, IL-8, IL-10, IL-15, and tumor necrosis factor (TNF)—were found to be significantly higher in the sarcopenia group (Park et al., 2026). A study conducted on patients with hepatocellular carcinoma stated that serum IL-6 levels showed a significant negative correlation with the psoas muscle index (Choi et al., 2020). In contrast to this study, a meta-analysis including 17 studies and 11,249 participants (3072 sarcopenic patients and 8177 non-sarcopenic patients) revealed that serum IL-6 levels were not significantly different in individuals with sarcopenia compared to controls (Bano et al., 2017). In our study, a negative correlation was also observed between SMI and IL-6 levels, but it did not reach statistical significance. Furthermore, IL-6 was found to be statistically significant in univariate analysis but not significant in multivariate analysis (p = 0.086). The reasons for the conflicting results in the literature include the failure to fully evaluate sarcopenia using the current definition, differences in the methods used across studies, different cut-off values applied for sarcopenia, and the heterogeneity of the study populations. In addition, different mechanisms may be involved in cancer patients, which may lead to different results.

Circulating IGF-1 levels are known to decrease in cachectic cancer patients (Morissette et al., 2009). A study by Zhuang et al. demonstrated that circulating IGF-1 levels, as well as muscle IGF-1-Ea and IGF-1-Eb transcript levels and muscle IGF-1 protein content, were reduced in animals with cachectic cancer (Bonetto et al., 2013; Zhuang et al., 2016). In another study, vastus lateralis muscle biopsies were performed on 55 hemodialysis patients and 21 healthy individuals to examine growth factors, including IGF-I. As expected, mRNA for IGF-I/IGF-I receptor in skeletal muscle were decreased in hemodialysis patients compared with healthy controls, whereas muscle IGF-I and serum IGF-II protein levels were increased (Wang et al., 2005). A total of 130 rats with hepatoma showed lower IGF-1 levels in the liver and skeletal muscle, suggesting that impaired IGF-1 signaling plays a role in the pathogenesis of muscle loss in tumor-bearing rats (Costelli et al., 2006). In a cross-sectional, multicenter study conducted in patients with type 1 diabetes (n = 1328), Hata et al. investigated IGF-1 levels and the relationship between IGF-1 and sarcopenia. They found that decreased serum IGF-1 was independently associated with sarcopenia and low SMI (Hata et al., 2021).

In our study, consistent with the literature, IGF-1 levels were lower in sarcopenic patients compared to non-sarcopenic patients. In our literature review, IGF-1 has not been studied as a marker for sarcopenia in lung cancer, making our study unique in this regard. Further studies including larger numbers of patients and different cancer types will provide additional support for our findings.

Myostatin is produced by muscle and inhibits muscle anabolism and satellite cell synthesis (Lee, 2021). It has been associated with decreases in muscle mass. A study in rats reported that the administration of myostatin resulted in significant muscle loss in rats (Costelli et al., 2008). Yaresheski et al. investigated myostatin levels in 58 patients with age-related muscle wasting and showed that mean serum myostatin levels increased with age (Yarasheski et al., 2002). A study in patients with liver cirrhosis revealed that high serum myostatin levels were negatively correlated with the psoas muscle index and associated with decreased overall survival rates (Nishikawa et al., 2017). Kawaguchi et al. evaluated sarcopenia and myostatin expression in 148 patients with non-small cell lung cancer (NSCLC). They reported that myostatin levels were statistically significantly higher in sarcopenic patients (Kawaguchi et al., 2024). In another study emphasized that high myostatin expression in lung cancer was significantly associated with low skeletal muscle mass and poor survival (Kawaguchi et al., 2024). Contrary to these studies exhibiting increased myostatin levels with sarcopenia, some studies reported a decrease in myostatin levels (Choi et al., 2020; Loumaye et al., 2015; Orioli et al., 2024). As an example of studies indicating decreased myostatin levels in sarcopenic patients, Choi et al. reported in a prospective study involving 238 patients with hepatocellular carcinoma that serum myostatin levels were decreased in cancer cachexia and showed a positive correlation with muscle mass (Choi et al., 2020). Supporting this result, another study involving 152 cancer patients found that plasma myostatin concentrations were reduced in sarcopenic patients compared to non-sarcopenic patients, and a positive correlation was found between muscle mass index and plasma myostatin levels (Loumaye et al., 2015). In prospective study involving 143 with cancer cachexia patients, Orioli et al. found that myostatin were significant for muscle mass, and correlation analysis revealed a positive correlation between SMI and myostatin (Orioli et al., 2024).

In our study, despite conflicting results in the literature, serum myostatin levels increased with sarcopenia, and SMI and HGS showed a negative correlation with myostatin levels. Due to myostatin’s relationship with muscle atrophy and its role as a primary negative regulator of muscle growth (Peng et al., 2018), an increase in its levels is expected in sarcopenia. The paradoxical findings in some studies suggest that myostatin may be downregulated in certain cases due to reduced muscle mass associated with sarcopenia.

When linear regression analysis was performed between HGS and myostatin, IL-6, and IGF-1, myostatin and IL-6 were found to be statistically significant. IGF-1 was found to be inadequate in predicting skeletal muscle strength. In the linear regression analysis between SMI and myostatin, IGF-1, and IL-6, myostatin was found to be statistically significant, while IL-6 and IGF-1 were found to be inadequate in predicting muscle mass.

A comparison of ECOG:0 and ECOG:1/2 patients in terms of sarcopenia prevalence showed that the sarcopenia rate was lower in the ECOG:0 group. In a study by Oflazoğlu et al. including cancer patients, the incidence of sarcopenia was associated with a worse ECOG performance score (Oflazoglu et al., 2020). In a study defining sarcopenia by measuring the psoas muscle index using computed tomography which included 286 hemodialysis patients, the psoas muscle index was higher in patients categorized as ECOG-PS:0 compared with thaose categorized as ECOG-PS: 2/3 (Kitamura et al., 2021). Poorer physical performance may lead to less physical activity and lower nutritional intake, which may ultimately result in muscle mass loss. According to this hypothesis, the prevalence of sarcopenia may be higher in the group of patients with lower performance scores.

Studies in the literature indicate that the incidence of sarcopenia is lower in obese and overweight patients than in normal weight and underweight patients. In a study including 191 elderly patients, Merchant et al. observed a lower incidence of sarcopenia in patients with a high BMI (Merchant et al., 2021). Curtis et al. in the ELSA cohort (n:5783) examined BMI and sarcopenia in older adults. They reported that low BMI was significantly associated with an increased likelihood of sarcopenia in a large cohort of community-dwelling older adults, representing a group at high risk for screening and intervention (Curtis et al., 2023). Morlino et al. reported in a study of 122 patients with breast cancer investigating the prevalence of sarcopenia that 66 (56%) had a BMI between 18.5 and 24.9 kg/m^2^, while 56 (46%) had a BMI between 25 and 30 kg/m^2^ (Morlino et al., 2022). In a retrospective study, Tanaka et al. examined physical performance as a prognostic indicator and paraspinal muscle sarcopenia in 587 patients with non-small cell lung cancer. BMI <18.5 kg/m^2^ was associated with a higher likelihood of sarcopenia and a positive short distances on the 6-min walk test. In the same study, the presence of diabetes was not significantly associated with sarcopenia (Tanaka et al., 2021). In contrast, de Carvalho et al. showed in a large population-based cohort of 5,181 older adults that older men with abdominal obesity had a faster rate of decline in muscle strength over an 8-year follow-up, despite having greater muscle strength at baseline (de Carvalho et al., 2019). In our study, no statistically significant difference was found between BMI and sarcopenia status. This may be related to the small sample size, the presence of advanced metastatic disease in our cohort, and the categorization of our BMI measurements as above or below 25 kg/m^2^. Furthermore, the lack of data on patients’ BMI values prior to the diagnosis of malignancy is a limitation of our study. However, clinicians should be aware that sarcopenia may also occur in patients with malignancy who have a normal BMI. At the same time, the lack of clear differentiation between fat mass and muscle mass during BMI measurement may lead to paradoxical results.

Kim et al. found a statistically significant association between serum albumin levels and sarcopenia in a study of 186 patients with small cell lung cancer, which examined the relationship between sarcopenia and the inflammatory response (Kim et al., 2016). In our study, patients with low albumin levels were numerically more prevalent in the sarcopenic patient group; however, this difference was not statistically significant. In the present study, we used an albumin cut-off level of 3.5 g/dL and categorized patients accordingly; however, albumin levels were moderately lower in sarcopenic patients than in non-sarcopenic patients (The albumin level was 3.9 g/dL in sarcopenic patients, and the albumin average of non-sarcopenic patients was 4.1 g/dL (p:0.084)). Furthermore, although a numerical difference was observed in our study, the lack of statistical significance may be due to the albumin cut-off value used and the small sample size.

Although the prevalence of sarcopenia increases with age, as reported in the literature (Goral, 2016; Sun et al., 2026), no statistically significant association between sarcopenia and age was observed in our study. This may be because, in our study, we compared only patients over 65 years of age with those under 65 years of age, and the mean ages of the patient groups were similar; thus, this finding likely reflects a cross-sectional comparative difference and does not rule out an age-related increase in the prevalence of sarcopenia. Furthermore, since our patient group consisted of patients with metastatic lung cancer, the findings may not fully reflect those reported in the literature due to the relatively shorter overall survival time and the similarity of the patient groups. Our study is unique in that it is a cross-sectional, prospective study with a sufficient number of patients, focuses on a specific cancer type, is evaluated using the currently accepted definition and measurement method for sarcopenia, and these markers have not been previously examined in lung cancer patients using this measurement method that defines sarcopenia. Our study also had some limitations. Firstly, it was a single-center study, and the majority of our patients were male, which prevented us from determining whether the parameters examined would yield different results based on sex. This may be due to the higher prevalence of lung cancer in the male population and the lower prevalence of sarcopenia in women, which may have contributed to the lack of sex balance among patients. Secondly, although the lack of consensus on cut-off values for HGS and SMI measurements in oncology is a limitation, the threshold values recommended by established guidelines for non-cancer populations were used as a basis. Finally, although computed tomography is recommended as the gold standard for measuring muscle mass index, BIA was preferred in this study due to its lower cost, lack of requirement for specialized personnel, and absence of radiation exposure for patients. Furthermore, BIA is among the tools recommended by guidelines for SMI measurement.

## 5 Conclusion

Our study expands the existing literature by simultaneously investigating both the quantitative and qualitative aspects of muscle mass. Furthermore, we conclude a correlation between sarcopenia developing in cancer patients and myostatin, IGF-1 and IL-6 levels. This correlation has the potential to shed light on future studies aimed at revealing its prognostic and predictive potential. We also believe these findings possess the potential to encourage future prospective studies exploring the clinical relevance of using myostatin, IGF-1, and IL-6 as early indicators of sarcopenia.

## Data Availability

The raw data supporting the conclusions of this article will be made available by the authors, without undue reservation.
